# A reference linkage map for *Eucalyptus*

**DOI:** 10.1186/1471-2164-13-240

**Published:** 2012-06-15

**Authors:** Corey J Hudson, Jules S Freeman, Anand RK Kullan, César D Petroli, Carolina P Sansaloni, Andrzej Kilian, Frank Detering, Dario Grattapaglia, Brad M Potts, Alexander A Myburg, René E Vaillancourt

**Affiliations:** 1 School of Plant Science and CRC for Forestry, University of Tasmania, Private Bag 55 Hobart, Tasmania, 7001, Australia; 2 CRN Research Fellow, Faculty of Science, Health, Education and Engineering, University of the Sunshine Coast, Locked Bag 4, Maroochydore, QLD, 4558, Australia; 3 Department of Genetics, Forestry and Agricultural Biotechnology Institute (FABI), University of Pretoria, Pretoria, 0002, South Africa; 4 EMBRAPA Genetic Resources and Biotechnology - EPqB Final W5 Norte 70770–917 Brazilia DF and Dep. Cell Biology, Universidade de Brazilia – UnB, Brasilia, DF, Brazil; 5 Diversity Arrays Technology Pty Ltd, PO Box 7141, Yarralumla, ACT, 2600, Australia; 6 EMBRAPA Genetic Resources and Biotechnology - Parque Estação Biológica - PqEB - Av. W5 Norte (final), Brasília, DF - Brazil - 70770–917, Universidade Catolica de Brasília- SGAN, 916 modulo B, 70790-160, DF, Brasilia, Brazil

## Abstract

**Background:**

Genetic linkage maps are invaluable resources in plant research. They provide a key tool for many genetic applications including: mapping quantitative trait loci (QTL); comparative mapping; identifying unlinked (*i.e.* independent) DNA markers for fingerprinting, population genetics and phylogenetics; assisting genome sequence assembly; relating physical and recombination distances along the genome and map-based cloning of genes. Eucalypts are the dominant tree species in most Australian ecosystems and of economic importance globally as plantation trees. The genome sequence of *E. grandis* has recently been released providing unprecedented opportunities for genetic and genomic research in the genus. A robust reference linkage map containing sequence-based molecular markers is needed to capitalise on this resource. Several high density linkage maps have recently been constructed for the main commercial forestry species in the genus (*E. grandis*, *E. urophylla* and *E. globulus*) using sequenced Diversity Arrays Technology (DArT) and microsatellite markers. To provide a single reference linkage map for eucalypts a composite map was produced through the integration of data from seven independent mapping experiments (1950 individuals) using a marker-merging method.

**Results:**

The composite map totalled 1107 cM and contained 4101 markers; comprising 3880 DArT, 213 microsatellite and eight candidate genes. Eighty-one DArT markers were mapped to two or more linkage groups, resulting in the 4101 markers being mapped to 4191 map positions. Approximately 13% of DArT markers mapped to identical map positions, thus the composite map contained 3634 unique loci at an average interval of 0.31 cM.

**Conclusion:**

The composite map represents the most saturated linkage map yet produced in *Eucalyptus.* As the majority of DArT markers contained on the map have been sequenced, the map provides a direct link to the *E. grandis* genome sequence and will serve as an important reference for progressing eucalypt research.

## Background

Genetic linkage maps are valuable resources which can be used to provide a framework for many genomic analyses. Linkage maps can be used to investigate the organisation and evolution of genomes through comparative mapping [1-3] and serve as a basis for investigating phenotypic traits of ecological and economic importance through the localisation of quantitative trait loci [QTL; [4-6]. Subsequently, QTL results may be used to help guide the selection of candidate genes for association studies or be applied in marker-assisted breeding programmes [7,8]. Linkage maps can also be used to anchor physical maps and assist in the assembly of genome sequences [9-11]. The wide application of linkage maps in combination with their value to genetics research has led to numerous linkage mapping projects being undertaken in plants. Detailed linkage maps have been produced for all of the world’s staple cereal species [12], and in forest trees, linkage maps have been produced for many of the most widely-planted species due to their commercial importance as wood and fibre crops [1,13,14].

Grattapaglia and Sederoff [15] published the first genetic linkage map in the forest tree genus *Eucalyptus* in 1994. Subsequently, many mapping pedigrees have been established for the purpose of linkage map construction and associated QTL analyses. More than 20 eucalypt genetic linkage maps have been reported with most being produced in the main commercially grown species, or their hybrids, from the *Eucalyptus* subgenus *Symphyomyrtus.* Thus, the majority of linkage mapping projects have focussed on *E. grandisE. urophylla* and *E. globulus* [reviewed in [16], while a smaller number of maps have also been produced for *E. nitens*[17], *E. teriticornis*[18,19], *E. camaldulensis*[20] and for species in the closely related genus *Corymbia*[21].

Many early eucalypt linkage maps were constructed using random amplification of polymorphic DNA (RAPD) and amplified fragment length polymorphism (AFLP) molecular markers [16,22]. However, the anonymous nature of these dominant markers has limited the transfer of linkage information between studies [16,23]. More informative, codominant markers such as isozyme and random fragment length polymorphism (RFLPs) have also been used in eucalypt linkage mapping, although, their low throughput, low inter-pedigree polymorphism and labour intensive genotyping requirements have limited their use [16,23]. The more recent development of highly polymorphic microsatellite markers made available a large potential suite of markers that are transferrable between species and polymorphic in multiple pedigrees. This enabled linkage group synteny to be established between maps containing common microsatellite markers and the positions and stability of QTL across multiple species to be examined [e.g. [24-27]. The ability to establish linkage group synteny has also enabled moderate-density comparative mapping studies [23,28].

Recent advances in molecular methods have led to high-throughput genotyping systems being developed [e.g. [29,30]. These have made it possible to quickly generate many hundreds of markers in single mapping pedigrees and have helped facilitate the construction of high density linkage maps [12]. Most recently in *Eucalyptus*, Diversity Arrays Technology [DArT; [31] has been used to generate large numbers of molecular markers for genetic linkage mapping in several mapping pedigrees [e.g. [11,32,33]. The eucalypt DArT markers are highly transferable across species from subgenus *Symphyomyrtus*[34] and the high-throughput array-based genotyping system provides wide genome coverage [35]. A key benefit of the *Eucalyptus* DArT markers is the public availability of the sequences of most of the 7680 markers contained on the genotyping array [GenBank accession numbers HR865291 - HR872186], thus making it possible to anchor DArT markers directly to the reference *E. grandis* genome sequence [v1.0 released January 2011; [36]. However, while the DArT technology offers many advantages, the DArT markers do suffer some limitations due to their dominant nature. For example, the incomplete segregation information provided by those DArT markers segregating in a 3:1 ratio (intercross) results in an exponential increase of marker-ordering calculations compared to fully-informative co-dominant markers [37]. Co-dominant markers also provide more complete information in QTL mapping studies [e.g. allowing estimation of additive and dominant allelic effects; [38] and are more useful in some genetic analyses, such as estimating population genetic parameters (e.g. inbreeding levels), relative to dominant marker types such as DArT. In addition, the DArT marker assay can be subject to cross-hybridization from duplicated loci in the genome, although most such artifacts can be excluded by preselecting markers exhibiting Mendelian segregation ratios in mapping pedigrees.

At present, DArT markers have been used to construct linkage maps in seven independent *E. globulus* and/or *E. grandis* × *E. urophylla* hybrid family mapping pedigrees [11,32,33]. All of these maps also contain a variable number of co-dominant microsatellite markers, which provide important links to many earlier eucalypt linkage maps. In the two largest mapping pedigrees (more than 500 individuals each), 1010 [32] and 2229 [33] DArT markers, were mapped at sub-centiMorgan marker densities and collectively more than 4000 DArT and microsatellite markers have been mapped in the seven pedigrees.

All DArT marker based linkage maps were constructed using the program JoinMap 4.0 [37]. This program is one of the most commonly used linkage mapping programs and appears to be the only software available for building linkage maps using the combined segregation data from multiple populations [39-41]. However, it is presently not feasible to combine the segregation data contained within the seven eucalypt mapping families describe above (collectively 1950 individuals), and successfully order such large numbers of markers within linkage groups (up to ~ 500) due to computational limitations (Van Ooijen *pers comm.*). To circumvent the limitations of traditional segregation-based methods of linkage map construction, alternative marker-merging strategies have been developed. A so-called ‘composite map’ can be produced in which markers from individual component maps are merged into a single map based on their position relative to common anchor loci. For example, the ‘neighbours’ marker-merging approach of Cone *et al*. [42] and the marker-merging method implemented in the PhenoMap program (GeneFlow Inc. USA) have been used to successfully construct high density composite maps containing several thousand markers in a number of plant species; including *Sorghum*[43], barley [41,44,45] and maize [42,46].

In this study, a marker-merging method was used to construct a high-density DArT and microsatellite marker composite linkage map from seven independently constructed maps. Recent comparative mapping analyses using 236 to 393 markers shared between three of the maps [see [32] showed that these linkage maps exhibited high synteny (> 93.4% markers occurring on the same linkage groups) and high colinearity (> 93.7% markers having the same order within linkage groups). This indicated that it would be possible to merge markers from several component maps into a single high quality map featuring robust marker-order together with very high marker density. It is expected that this composite map will facilitate marker and map information exchange and serve as a valuable reference for species in the subgenus *Symphyomyrtus*.

## Methods

The following terms are used to describe the various types of linkage maps reported in this paper; (1) sex-averaged map – a consensus of individually constructed male and female maps, built in a single family using segregation data from both parents, (2) consensus map – a consensus of multiple individually constructed male and female maps, built in multiple families (e.g. F_2_ double-pseudo backcross) using segregation data from all of the families, and (3) composite map – an integrated map of multiple sex-averaged and/or consensus maps, built using a marker-merging method.

### Component maps

The composite map was built using an *E. grandis* × *E. urophylla* F_2_ double pseudo-backcross pedigree consensus linkage map [both species from section *Latoangulatae*; [33] plus one *E. grandis* × *E. urophylla* sex-averaged map constructed in a F_1_ hybrid pedigree [11] and five pure-species *E. globulus* [section *Maidenaria*; [32] sex-averaged linkage maps constructed in either outcrossed F_2_ or F_1_ families (hereafter referred to as ‘component’ maps). Component map family sizes ranged from 172 (GLOB-F_2_-1) to 547 (GU-SA) and collectively contained 1,950 individuals (Table 1). The component maps were constructed by different researchers. All used JoinMap 4.0 [37] with marker-ordering within linkage groups (LGs) estimated using the regression algorithm of Stam [47] combined with the Kosambi mapping function. All component maps comprised 11 linkage groups in accordance with the haploid chromosome number of *Eucalyptus*[48].

**Table 1:** Component map details

**Linkage map^a^**	**Map abbreviation**	***n***	**cM**	**MMI**	**Markers mapped (percentage of unique markers in pedigree)**			
					**DArT**	**SSR**	Gene	**Total**
*E. grandis* × *E. urophylla* SA double pseudo-backcross F_2_^b^	GU-SA	547	1107	0.51	2229 (45%)	59 (46%)	2 (100%)	2290 (45%)
*E. grandis* × *E. urophylla* Embrapa F_1_^ce^	GU-Emb	177	1229	0.78	1617 (41%)	193 (77%)	0	1810 (44%)
*E. globulus* Lighthouse F_2_^d^	GLOB-LH	503	1151	1.21	1010 (27%)	50 (12%)	0	1060 (27%)
*E. globulus* FAM1 F_1_^d^	GLOB-F_1_-1	184	1033	1.97	571 (14%)	4 (0%)	2 (0%)	577 (14%)
*E. globulus* FAM4 F_1_^d^	GLOB-F_1_-4	184	1137	2.46	488 (10%)	6 (0%)	4 (25%)	498 (10%)
*E. globulus* FAM5 F_1_^d^	GLOB-F_1_-5	183	1055	2.09	600 (22%)	4 (0%)	2 (0%)	606 (21%)
*E. globulus* FAM1 F_2_^d^	GLOB-F_2_-1	172	1258	2.73	660 (18%)	30 (30%)	5 (40%)	695 (18%)

Summary of the component maps used to construct the composite map. For each map, progeny size (*n*), map length (cM; total for all 11 linkage groups), mean marker interval (MMI; average for all 11 linkage groups) and total number of mapped markers (using only those linkage groups included in composite map construction; see Methods) are given. For DArT, microsatellite (SSR) and gene markers mapped on each component map, the percentage of markers unique to that map (*i.e.* not mapped in any of the six other component maps) are given in parentheses. ^a^Cross details and reference; ^b^Kullan *et al.*[33], ^c^Petroli *et al.*[11] and ^d^Hudson *et al.*[32]. ^e^Data for the *E. grandis* × *E. urophylla* Embrapa F_2_ component map calculated using a combination of framework and comprehensive linkage groups (see Methods).

Before building the composite map, marker names were standardised across maps, homologous linkage groups were identified using common (anchor) loci and marker colinearity between component maps was visually inspected in MapChart [49]. Map data was supplied for both framework (1032-marker) and comprehensive (2484-marker) maps built in the GU-Emb family [see [11]. Based on the level of marker-order agreement between linkage groups from these maps with other component maps, either GU-Emb framework (LG’s 1, 3, 5, 7 and 9) or comprehensive (LG’s 2, 4, 6, 8, 10 and 11) linkage groups were included in composite map construction. Five linkage groups from three of the smaller *E. globulus* mapping families (Table 1) were found to have substantial regions of non-colinearity (discordant marker-orders) with other component maps. Consequently, LG6 and LG10 from the GLOB-F_1_-1 map, LG4 and LG9 from the GLOB-F_1_-4 and LG4 from the GLOB-F_1_-5 map were excluded from composite map construction.

The number of markers included for composite map construction ranged from 498 (GLOB-F_1_-4) to 2290 (GU-SA; Table 1). In total, this consisted of 4350 individual markers, including: 4089 DArT, 253 microsatellites and eight mapped genes. Ninety-six markers (2.2% of the total number of markers; termed ‘multicopy’ markers) were mapped to two or more linkage groups across component maps. This resulted in the 4350 individual markers being mapped to 4457 positions. Of these 4457 positions, 1960 could be considered to be bridging loci, meaning that these markers had been mapped to syntenic linkage groups in two or more component maps and would serve as anchor loci during composite map construction. Conversely, 2497 marker positions were unique to single component maps.

### Composite map construction

The composite linkage map was constructed at Diversity Arrays Technology (DArT) Pty Ltd (Canberra, Australia) using specially developed R scripts which merged component map markers into the composite map based on their relative map positions. The *E. grandis* × *E. urophylla* SA F_2_ (GU-SA) linkage map was used as the seed-map (*i.e.* the ‘fixed backbone’ to which markers from other component maps were added) due to it having the largest progeny size, the largest number of both mapped and unique markers (Table 1) and high overall marker colinearity to the 11 main superscaffolds of the assembled *E. grandis* genome sequence [33,36]. The procedure for building each composite map linkage group was as follows. Firstly, the number of common markers in each seed-map – component map linkage group comparison was identified. Spearman rank marker-order correlations were then estimated and a heuristic ‘fit value’ for each comparison was calculated as; Fit value = correlation × log (number of common markers); where the second term rewards for the number of common markers with a diminishing returns function. Following selection of the component map linkage group with the highest Fit value, unique markers (*i.e.* those not mapped on the seed linkage group, or the ‘building’ composite linkage group in following rounds) were added to the seed linkage group (or ‘building’ composite map linkage group) using linear regression. Here, the slope (m) and intersect (c) calculated from fitting the positions of common markers on the seed linkage group (pc) to their positions on the selected component map (pi) linkage group (pc = m × pi + c) was used to calculate the positions of unique component map markers added to the seed linkage group. Once this first round was completed, the remaining component linkage groups were compared to this new ‘building’ composite map linkage group and the process was repeated. This continued until all unique markers had been added from remaining component maps which shared at least three common markers with the building composite map linkage group and had a marker-order correlation coefficient ≥ 0.50. This process was repeated for each linkage group to yield the final composite map of 11 linkage groups. Markers which mapped to the distal ends of composite linkage groups and which had relatively large inter-marker intervals (≥ 5 cM) and poor support (e.g. mapped in one component map only) were removed. The numbering and orientation of linkage groups followed the convention established in Brondani *et al.*[23]; this also corresponds to the numbering of pseudochromosome assemblies in the *E. grandis* genome sequence [36].

### Composite map features

Following composite map construction, marker-order correlations between composite and component map linkage groups were calculated in SAS 9.2 (SAS Institute, Cary, USA) using the PROC CORR Spearman function. To test whether multicopy markers were distributed equally across linkage groups, a *χ*^2^ test was used to compare the observed versus expected number of multicopy marker positions occurring on each linkage group. The expected number of multicopy markers per linkage group was calculated as; (total number of multicopy marker positions in the composite map/total number of DArT marker positions in the composite map) × number of DArT marker positions per linkage group for that linkage group. The BLAST server available at Phytozome [36] was used to search for DArT marker duplications. The bl2seq tool at NCBI [50] was used to examine DArT marker sequence similarity/redundancy. All graphical representations of linkage maps were drawn using MapChart [49].

## Results

### Composite map details

A total of 4101 individual markers, comprising 3880 DArT markers, eight gene-based markers and 213 microsatellite markers were included in the composite map. The composite map totalled 1107 cM which was within the range of component map lengths (1033–1258 cM; Table 1) and contained only eleven marker intervals ≥ 3 cM; with a maximum marker interval of 5.9 cM. The composite map contained 81 multicopy DArT markers (2.1% of total DArT markers) which were mapped to 171 map positions. Most multicopy markers occurred on two linkage groups only, however, one marker (ePt-574238) mapped to three linkage groups while four markers (ePt-503174, ePt-568818, ePt-637610, ePt-637861) mapped to four linkage groups. This resulted in the 4101 markers being mapped to 4191 positions (Table 2). Over half (2171 or 53%) of the markers mapped to these 4191 map positions had been mapped in a single component map only (*i.e.* were not shared among multiple component maps). Approximately 13% of DArT markers mapped to identical positions in the composite map. Therefore, the map contained 3634 unique map loci with an average interval of 0.31 cM.

**Table 2:** Composite map summary

**LG**	**cM**	**Markers mapped**				**Average marker interval (cM)**	**MC DArT pos.^a^**
				**DArT**	**SSR**	**Genes**	**Total**		
1	93.8	250	12	0	262	0.42	5
2	102.1	451	29	0	480	0.24	18
3	105.6	429	18	2	449	0.28	21
4	80.9	219	9	3	231	0.41	12
5	95.9	366	8	0	374	0.30	24
6	125.3	408	43	1	452	0.31	15
7	87.7	305	9	1	315	0.33	18
8	137.3	540	26	0	566	0.28	19
9	82.9	312	20	0	332	0.29	10
10	97.8	336	20	1	357	0.30	12
11	97.3	354	19	0	373	0.31	17
*Total*	1106.5	3970	213	8	4191	0.31	*171*^b^

Summary of the composite map: including the number of mapped markers, length and average marker intervals by linkage group (LG). ^a^MC DArT pos. - indicates the number of multicopy (MC) DArT marker positions (pos.) occurring on each linkage group. ^b^The 171 multicopy DArT marker positions represent 81 multicopy DArT markers (see Additional file 1).

The number of multicopy DArT marker positions on each linkage group ranged from 5 to 24 and represented 1.9-6.4% of the total number of DArT markers mapped per linkage group (Table 2). Although LG5 and LG7 contained a larger proportion of multicopy DArT marker positions (e.g. LG1 contained only 5 multicopy DArT marker positions, or 1.9% of the total number of DArT marker positions; Table 3), the proportion of multicopy DArT marker positions found on each linkage group did not significantly differ from that expected by chance across all linkage groups (*χ*^2^ = 12.99, *P* = 0.22, *df =* 10). There was no trend within linkage groups for multicopy DArT markers to be clumped in either distal or central linkage group areas (data not shown). Composite map marker details, component map(s) marker origins and multicopy DArT marker information is presented in Additional file 1.

**Table 3:** Composite – component map marker-order correlation coefficients

**Composite map LG**	**Component map**							
	**GLOB-LH**	**GU-Emb**	**GLOB-F_2_-1**	**GLOB-F_1_-1**	**GLOB-F_1_-4**	**GLOB-F_1_-5**	**Average^a^**	**Average^b^**
1	0.98*	0.56^F^	0.99*	0.99*	0.98*	0.95*	0.91	0.98
2	0.98*	0.95^C^*	0.93*	0.95*	0.98*	0.85	0.94	0.96
3	0.91*	0.99^F^*	0.97*	0.99*	0.97*	0.99*	0.97	0.97
4	0.98*	0.74^C^	0.96*	0.89	0.79^ex^	0.19^ns,^^ex^	0.76	0.97
5	0.99*	0.92^F^	0.96*	0.96*	0.99*	0.96*	0.96	0.97
6	0.99*	0.99^C^*	0.99*	0.63^ex^	0.99*	0.86	0.91	0.99
7	0.98*	0.65^F^	0.99*	0.98*	0.96*	0.91*	0.91	0.96
8	0.98*	0.99^C^*	0.99*	0.66	0.94*	0.99*	0.93	0.98
9	0.99*	0.97^F^*	0.98*	0.97*	0.65^ex^	0.97	0.92	0.98
10	0.95*	0.98^C^*	0.97*	0.35^ns,^^ex^	0.92	0.97*	0.86	0.97
11	0.99*	0.99^C^*	0.99*	0.97*	0.96	0.99*	0.98	0.99
Average^c^	0.98	0.88	0.97	0.85	0.92	0.87		
Average^d^	0.98	0.98	0.97	0.97	0.97	0.96		

Marker-order correlations between composite map and component map linkage groups; the GU-SA component map is not shown as this map was used as the seed-map (*i.e.* provided a fixed-order and all correlations were 1.0). For the GU-Emb component map, superscript letters indicates whether the framework (F) or comprehensive (C) linkage group was used in map construction. Component map linkage groups initially excluded from composite map construction are indicated by ^ex^ superscript. An asterisk following the correlation value indicates that marker-order information from the component map was incorporated during construction of the composite map linkage group. Apart from two correlations (indicated by ^ns^ superscript) all correlations were significant at α ≤ 0.05. Averages: ^a^calculated using all six component maps, ^b^calculated using only those linkage groups included in composite map construction (marked with an asterisk), ^c^calculated using all 11 linkage groups, ^d^calculated using only those linkage groups included in composite map construction (marked with an asterisk).

### Composite – component map colinearity

**Figure 1:**
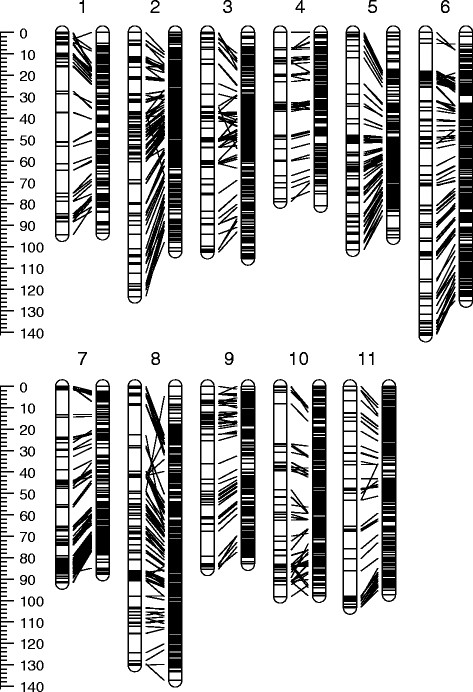
**Marker colinearity between the GLOB-LH component map (left) and composite map (right).** Lines between each homologous linkage group pair indicate the positions of common markers. The scale bar is in Kosambi’s centiMorgans.

Colinearity between component and composite map linkage groups can be viewed graphically in Figure 1 (for the GLOB-LH map) and in Additional file 2 (all component maps). Pair-wise linkage group marker-order correlations were generally high (greater than 0.90; Table 3) reflecting the high colinearity shown between common markers (Figure 1 and Additional file 2). However, a small degree of non-colinearity did occur between all component maps and the composite map. Eleven component map linkage groups had marker-order correlations of less than 0.90 (Table 3), however, these linkage groups were either, (1) identified as having poor marker colinearity with other component maps prior to composite map construction and excluded from analysis (five linkage groups with gray shading in Table 3), or (2) marker-order information from these linkage groups was not incorporated during composite map construction (correlation value without asterisk; six linkage groups Table 3) due to markers from these maps being previously added from other linkage groups having better fit values. Thus, these poorly correlated linkage groups did not adversely affect the composite map marker-order. For each linkage group, the average pair-wise marker-order correlation between the composite map and those component maps included in map construction ranged from 0.96 to 0.99 (Average^b^ column; Table 3).

### DArT marker duplications

Although not a main focus of this study, evidence for the occurrence of duplicated DArT marker loci within the assembled *E. grandis* genome sequence [36] was investigated for the five multicopy markers which had been mapped to three or more linkage groups. Two of these markers (ePt-637610 and ePt-637861; see Additional file 1) mapped to the same map position on each of four linkage groups (LGs 2, 3, 5 and 8) and were found to be redundant markers (*i.e.* identical sequences) based on their marker sequence similarity (bl2seq: 583/606 base-pair similarity, e-value: 0.0). For the four unique multicopy markers, three were detected to have loci duplications within the *E. grandis* genome sequence. In each case, the positions of duplicated loci detected in the *E. grandis* genome sequence corresponded to the linkage groups to which the marker was mapped.

## Discussion

### Composite map construction

Data from seven component maps were integrated into a single composite map which represents the highest density map yet produced in *Eucalyptus*. A major advantage of the marker-merger method used in this study was the substantial time and labour savings made when compared to the effort required to produce comparable maps using traditional, segregation-based methods. For example, Li *et al.*[40] constructed a 2111 marker composite map from four barley mapping pedigrees and reported that it took ‘*several thousand hours*’ of computing time. In a larger barley study, Wenzl *et al.*[41] produced a 2935 loci composite map from ten mapping populations using JoinMap 3.0 [51] in combination with specially built Perl scripts and reported that the project required several months of semi-manual data processing [41]. In contrast, the composite map produced in this study was built in a single day.

### Utility of the composite map

As sequences are available for the majority of DArT markers on the map (91%; data not shown), the composite map provides a direct link to the *E. grandis* genome sequence [36]. We have made use of this link to search the *E. grandis* genome sequence for candidate genes associated with QTL locations and to facilitate the placement of candidate genes in the component linkage maps without the need for time consuming marker development and genotyping. Sequence-based linkage maps have also provided useful tools to aid in the assembly of genome sequences [e.g. [52,53] and can be particularly beneficial in taxa (such as eucalypts) which have a relatively small genome size. For example, during the assembly of the *E. grandis* genome sequence, a DArT linkage map was valuable in guiding contigs into the 11 main pseudochromosomes [16]. However, not all contigs could be aligned and approximately 12% of the 693 Mbp *E. grandis* genome sequence remains unassembled in more than 4900 small unlinked scaffolds [54]. With the composite map containing many more DArT markers (1600+) than the linkage map used to aid genome assembly, the composite map markers may provide further positional information and help to anchor some of the unlinked scaffolds and refine the current *E. grandis* genome sequence.

Over half (53%) of the markers placed in the composite map originated from a single component map (*i.e.* were not shared among multiple component maps). Therefore, the ability to determine the relative positions of markers mapped in different maps has been greatly enhanced through the integration of this data into a single map. This has already proven advantageous to our research group, with the composite map being used to quickly identify the linkage relationships of microsatellite markers used in population genetic studies. Although now a relatively simple task, it was previously necessary to consult multiple linkage maps and assess their colinearity to obtain this same information. Furthermore, any marker developed in eucalypts which has known sequence, can now potentially be found in the eucalypt genome sequence and then aligned against the reference map in order to estimate its distance to other markers in units of recombination (cM); which are evolutionary meaningful units compared to base pair distances. Additionally, it is also important to understand the relationship between physical map (*i.e.* genome) and genetic map distances as this can have implications for map-based cloning efforts and/or marker-assisted selection. For example, uneven recombination rates across a genome [12,55] may result in physically distant markers appearing to be genetically close to each other, or vice versa. In eucalypts, Kullan *et al.*[33] recently compared 153 linkage map intervals of approximately 1 cM against contigs of the *E. grandis* genome and found that the genetic map to physical distance relationship varied considerably; ranging from 100 kb to 2.4 Mbp per 1 cM. Therefore, the composite map will be useful to provide further insight into the relationship between physical and genetic map distance in addition to identifying hot (or cold) spots of recombination.

A key use of the composite map will be for comparison of QTL and candidate gene positions detected across variable genetic backgrounds and/or environments in different studies. This has previously been limited due to a lack of common markers being shared between maps [23]. For example, Thumma *et al.*[27] detected multiple co-locating growth-related QTL on LG5 in *E. nitens* but could not accurately compare the position of this QTL to similar growth-related trait QTL detected on this same linkage group in two other studies [24,56]. Although most of the markers contained on the composite map are DArT markers, which to date have only been mapped in the pedigrees included in this study, the map does contain several hundred microsatellite markers (213) which will enable synteny and colinearity to be established with many earlier linkage maps used for QTL detection; e.g. 13 out of 22 earlier studies have mapped a variable number of microsatellites [16]. This will enable QTL to be aligned against the composite map which may provide deeper insight into the genetic control of phenotypic traits in the genus. For example, following the construction of an integrated map for melon (*Cucumis melo*) which used data from eight independent mapping experiments, it was possible to align 370 QTL detected for 62 traits from 18 experiments [57]. Through this alignment, QTL detected in different studies for economically important traits were found to co-locate [57]; providing supporting evidence to substantiate the biological basis of the observed marker-trait association [7,8].

As in all linkage mapping studies, it is important to consider both the quality of the map produced and any specific map characteristics. In the alignment of 6480 DArT marker sequences against the *E. grandis* genome sequence [36], Petroli *et al.*[11] reported that although the majority of markers (4189) occurred at a single genome position with high support, many marker sequences (2291), albeit at lower confidence, also exhibited similarity to a second genome position and that about half of these genome regions contained repeat elements. Furthermore, preliminary analysis of the *E. grandis* genome sequence suggests that (as has been observed in some *Rosid* genomes) a whole-genome duplication event has occurred in the lineage (*Myrtales*) subsequent to the ancient hexaploidy event shared by all rosids (Myburg *et al*., unpublished). Such whole-genome, as well as, segmental duplication events will affect thousands of marker loci, but most would be expected to diverge in sequence with evolutionary time yielding mostly unique marker loci. Thus, the presence of multicopy markers (representing putatively duplicated loci) in the composite map was not unexpected. It is worth noting that in the construction of each component map, only those markers which segregated as a single Mendelian locus were mapped. Therefore, in the event of a marker duplication being present within a pedigree, only one locus could be polymorphic in order for that marker to produce a single loci segregation ratios. Consequently, it is likely that only a subset of the duplicated loci present within the eucalypt genome have been identified in the composite map. Given that the *Pst*I enzyme used in the complexity reduction step of DArT marker development [35] preferentially produces markers located in hypomethylated, gene rich regions [55], and that many DArT markers contain protein coding sequences [33], it is possible that some of the multicopy markers identified may be associated with different gene family members and/or be part of larger duplicated regions. Further studies are required to examine the full extent and evolution of the duplicated loci. We also expected some marker redundancy (markers with the same sequence) among the 3808 composite map DArT markers; an issue which arises due to the process by which DArT markers are generated, resulting in the same amplified genomic fragment being represented more than once on the genotyping array [31,35]. Therefore, identical clones (e.g. the same DArT fragment, but with different DArT marker names) are expected to produce identical genotype scores and should map to identical (or near identical) map positions; as found for the markers ePt-637610 and ePt-637861 identified as identical clones in this study.

The marker-merging method used in this study took advantage of the fact that individual component maps were constructed using high marker-ordering stringency which resulted in linkage maps having robust marker-orders [32]. The comparison of the composite map marker-order against individual component maps gives an indication of the quality of the composite map. Marker-order correlations were mostly excellent with high pair-wise linkage group marker-order correlations found in most comparisons. For example, in 48 out of 66 pair-wise comparisons the marker-order correlation exceeded 0.95. Despite these high correlations, most component maps did exhibit some marker-order inconsistencies with the composite map. A number of (mostly) single marker-order inconsistencies did occur over large distances, but most marker-order disagreements occurred among tightly grouped markers in regions of less than 5 cM. Although it is possible that some of these marker-order differences could be real and represent local chromosomal rearrangements or marker duplications between the different mapping pedigrees and/or species, they are more likely to reflect marker-order inaccuracies within any of the component maps or simply be artefacts of the statistical uncertainty associated with ordering tightly linked markers [see [58]. While users of this map should be aware of these limitations and how they may affect marker ordering, overall, the generally high marker-order correlations observed and the exclusion of component map linkage groups having poor marker colinearity from initial composite map construction (and thus not adversely affecting composite map marker-order) suggests that the composite map is of a sufficiently high quality to facilitate the transfer of genetic information between studies.

The composite map will be most useful for studies involving species from subgenus *Symphyomyrtus* sections *Latoangulatae* and *Maidenaria*; due to the composite map being built from linkage maps constructed in species from these sections. However, due to the high level of genome synteny and colinearity detected between species from these relatively distant sections [28,32,34], information from the composite map should also be applicable to many other commercially important eucalypt species in closely related sections (e.g. *E. camaldulensis* from subgenus *Symphyomyrtus* section *Exsertaria*).

### Future marker integration

A number of recent studies have focussed on the development of molecular markers for use in eucalypts. In addition to the DArT genotyping array developed for use in eucalypts [35], the feasibility of high-throughput SNP genotyping has been explored [59] and several tens of species-transferrable EST-based SSR markers have been recently reported [60,61]. Furthermore, DArT genotyping by sequencing (GBS), which combines the complexity reduction method of DArT [31] with next generation sequencing (NGS), and which can potentially deliver up to three-fold as many markers as conventional DArT genotyping [see [62] is becoming a cost-competitive genotyping option due to the recent plummeting costs of NGS sequencing. Therefore, to broaden the use of the composite map for comparative analyses and to optimise its’ worth, it will be necessary to add new markers to the current version of the composite map in the future. Although beyond the scope of this study, it would also be valuable to compare the marker order of the composite map to maps built using the same data with other marker-merging software (e.g. BioMercator [63], CarthaGene [64] or MergeMap[65]). The R scripts and map marker positions of the component maps used in this study can be made available upon request.

## Conclusion

The integration of markers from seven individual genetic linkage pedigrees has resulted in a composite, reference map for eucalypts with 4101 DArT and microsatellite markers*.* Although some small marker-order inconsistencies exist between component maps and the composite map, there is a relatively high agreement of marker-order between component maps; which indicates that the composite map represents a good estimation of the true marker positions in most cases. However, at finer scales (sub-cM) marker-orders may differ between component and composite maps due to limited statistical power to order such tightly linked markers. Overall, the genome coverage and marker density of the composite map greatly exceeded that achieved in any of the single mapping populations. It is expected that this composite map will provide a valuable reference map for the world-wide *Eucalyptus* research community, facilitate the transfer of genetic information between different studies and allow for the integration of DArT marker information with other genomic resources.

## Competing interest

'The authors declare that they have no competing interests.

## Authors’ contributions

CJH built the GLOB-LH linkage map, coordinated the collection of map data, building of the composite map, performed analyses and wrote the manuscript. JSF built all other *E. globulus* linkage maps. AAM and ARKK, DG and CDP, built and contributed GU-SA and GU-Emb linkage maps, respectively. AK and FD constructed the composite map. REV and AAM conceived the study, and along with BMP and JSF, contributed to the design of the study. All authors read and approved the final manuscript.

## Funding

Funding for this project was provided by the Australian Research Council (DP0770506 & DP110101621) as well as the Cooperative Research Centre for Forestry (Australia). Construction of the GU-SA map was supported by Sappi, Mondi, the Technology and Human Resources for Industry Program (THRIP), the National Research Foundation (NRF) and the Department of Science and Technology (DST) of South Africa.

## Supplementary Material

Additional file 1 ***Eucalyptus*composite map details.**Details of markers mapped in the Eucalyptus composite map. Includes, linkage group and position of mapped markers, marker type and which component map(s) markers were mapped. A '1' in the 'Multicopy marker' column indicates that the marker occurs on two or more linkage groups. Click here for file

Additional file 2 **Composite map – component map marker colinearity.** Marker colinearity among all six component maps and the *Eucalyptus* composite map. For each linkage group, three linkage group ‘triplets’ show marker colinearity between two component maps (outside) and the composite map (centre). Horizontal lines on linkage group bars indicate marker positions and lines between linkage groups indicate the position of common markers. The scale bar shown is in Kosambi’s centiMorgans. Component map names (abbreviations; see Table 1) are given above each linkage group. Linkage groups excluded from composite map construction are indicated in parentheses following the component map name. An asterisk indicates whether marker-order information from the component map was incorporated during composite map construction (see Methods). For the GU-Emb component map, superscript letters indicates whether the framework (f) or comprehensive (c) linkage group from this pedigree was used in composite map construction. Click here for file
