# Supplementary material for: A reference linkage map for Eucalyptus
Source: BMC Genomics. 2012 Jun 15;13:240. doi: 10.1186/1471-2164-13-240 (PMC3436727; doi:10.1186/1471-2164-13-240)
Supplement: Additional file 2 — Composite map – component map marker colinearity. Marker colinearity among all six component maps and the Eucalyptus composite map. For each linkage group, three linkage group ‘triplets’ show marker colinearity between two component maps (outside) and the composite map (centre). Horizontal lines on linkage group bars indicate marker positions and lines between linkage groups indicate the position of common markers. The scale bar shown is in Kosambi’s centiMorgans. Component map names (abbreviations; see Table 1) are given above each linkage group. Linkage groups excluded from composite map construction are indicated in parentheses following the component map name. An asterisk indicates whether marker-order information from the component map was incorporated during composite map construction (see Methods). For the GU-Emb component map, superscript letters indicates whether the framework (f) or comprehensive (c) linkage group from this pedigree was used in composite map construction. [file 1471-2164-13-240-S2.pdf]

Additional file 2

Title: Composite map – component map marker colinearity

Description: Marker colinearity between all six component maps and the *Eucalyptus* composite map. For each LG, three LG 'triplets' show marker colinearity between two component maps (outside) and the composite map (centre). Horizontal lines on LG bars indicate marker positions and lines between LGs indicate the position of common markers. The scale bar shown is in Kosambi's centiMorgans. Component map names (abbreviations; see Table 1) are given above each LG. LGs excluded from composite map construction are indicated in parentheses following the component map name. An asterisk indicates whether marker-order information from the component map was incorporated during composite map construction (see Methods). For the GU-Emb component map, superscript letters indicates whether the framework (f) or comprehensive (c) linkage group from this pedigree was used in composite map construction.

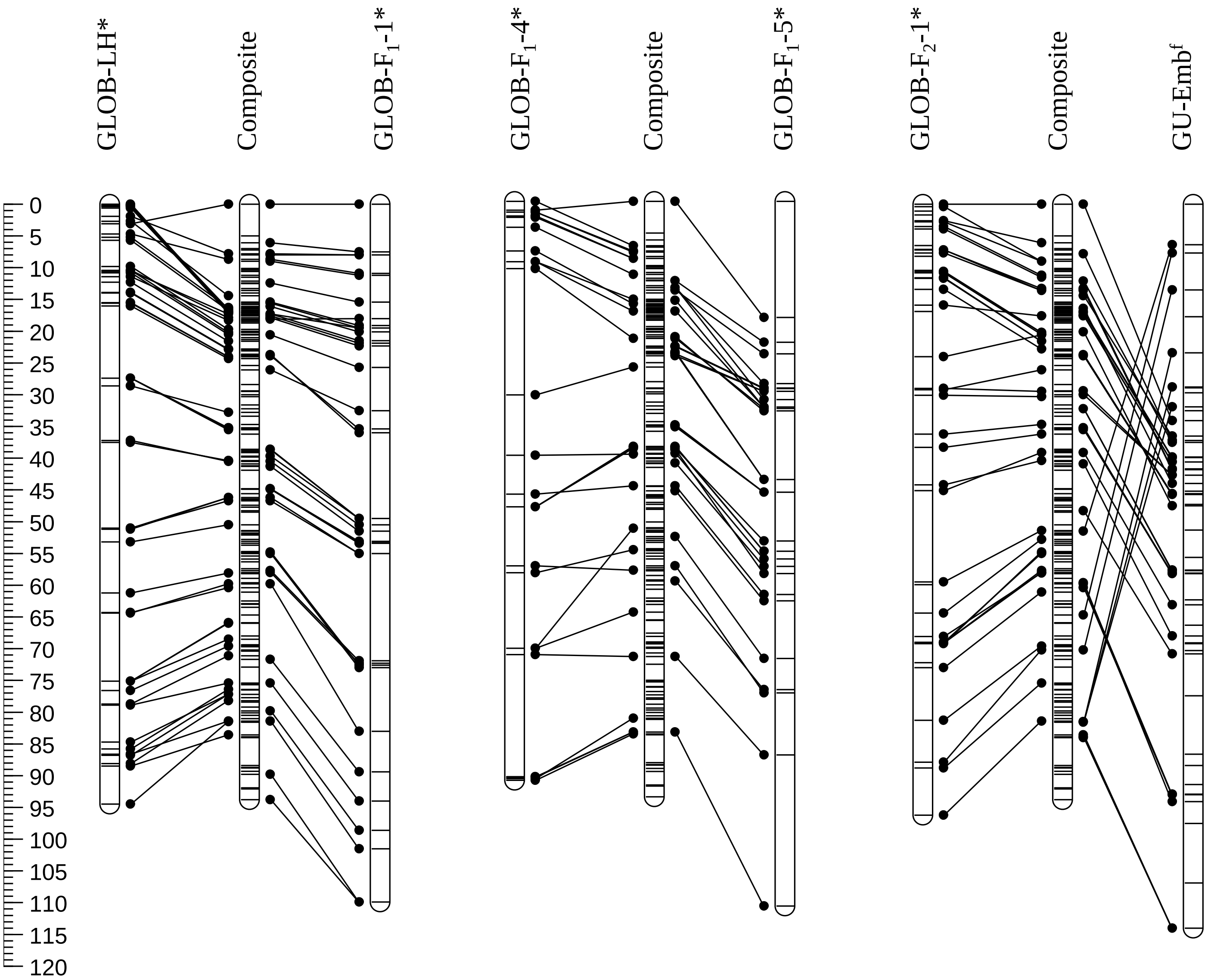

Linkage group 1

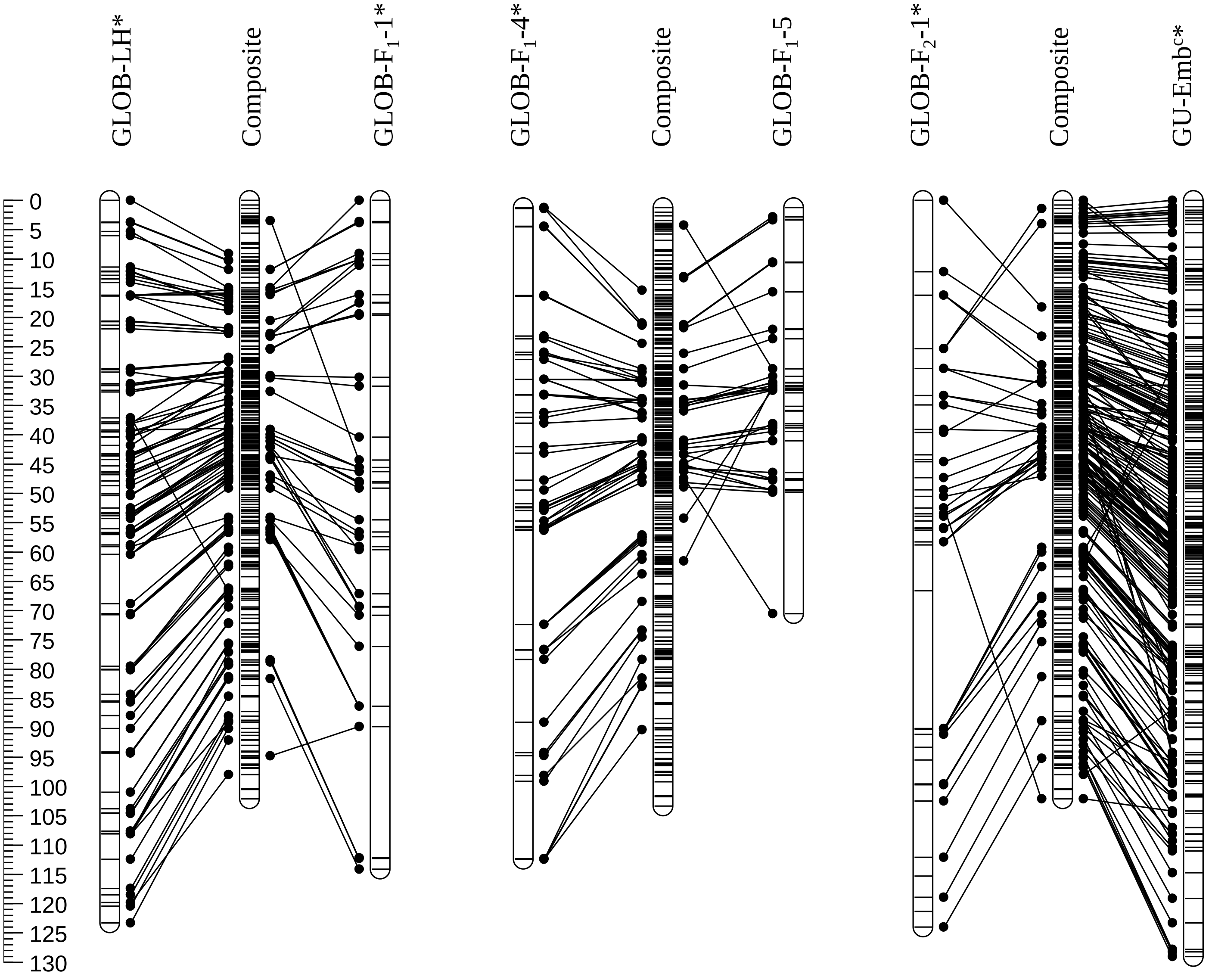

Linkage group 2

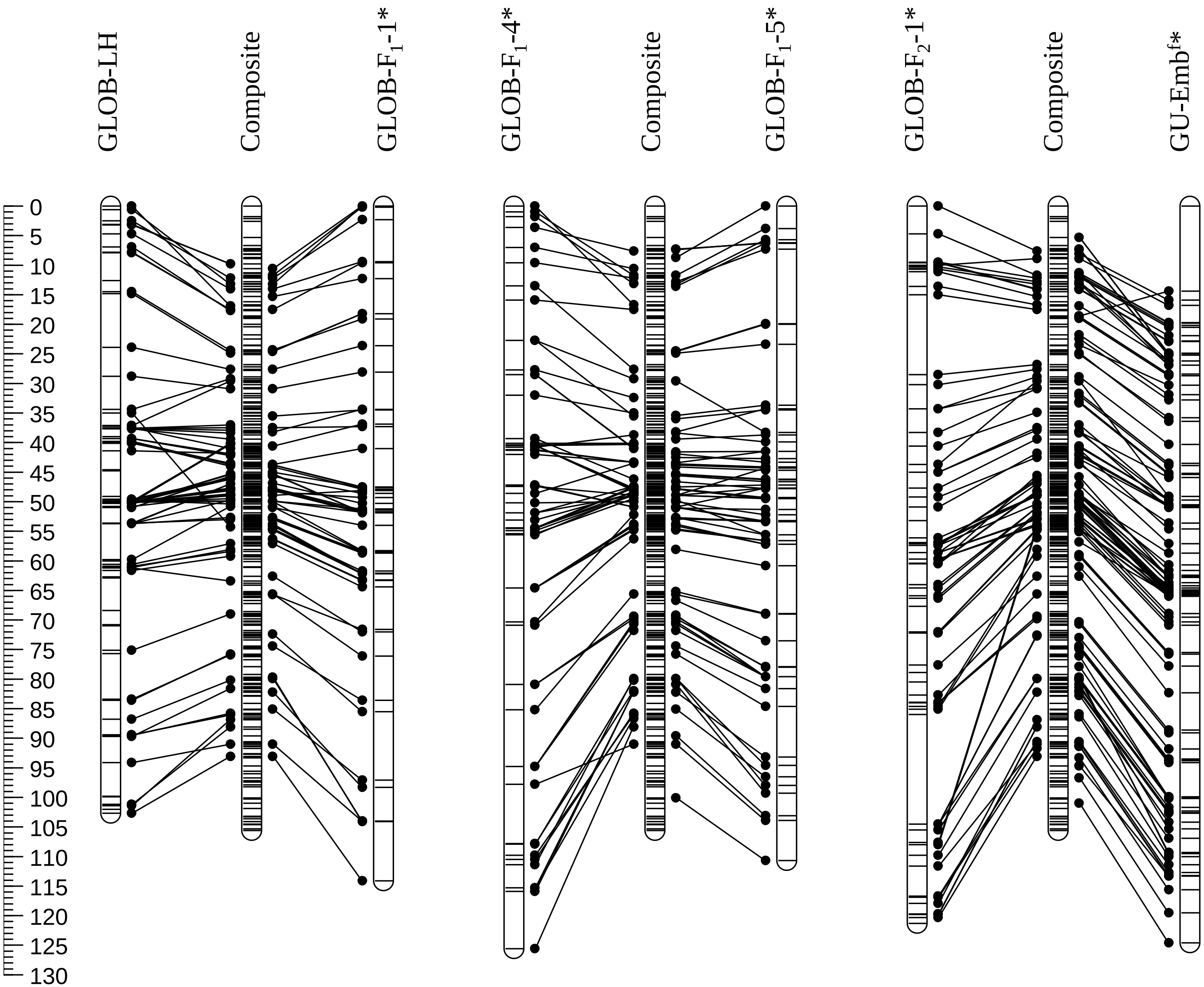

Linkage group 3

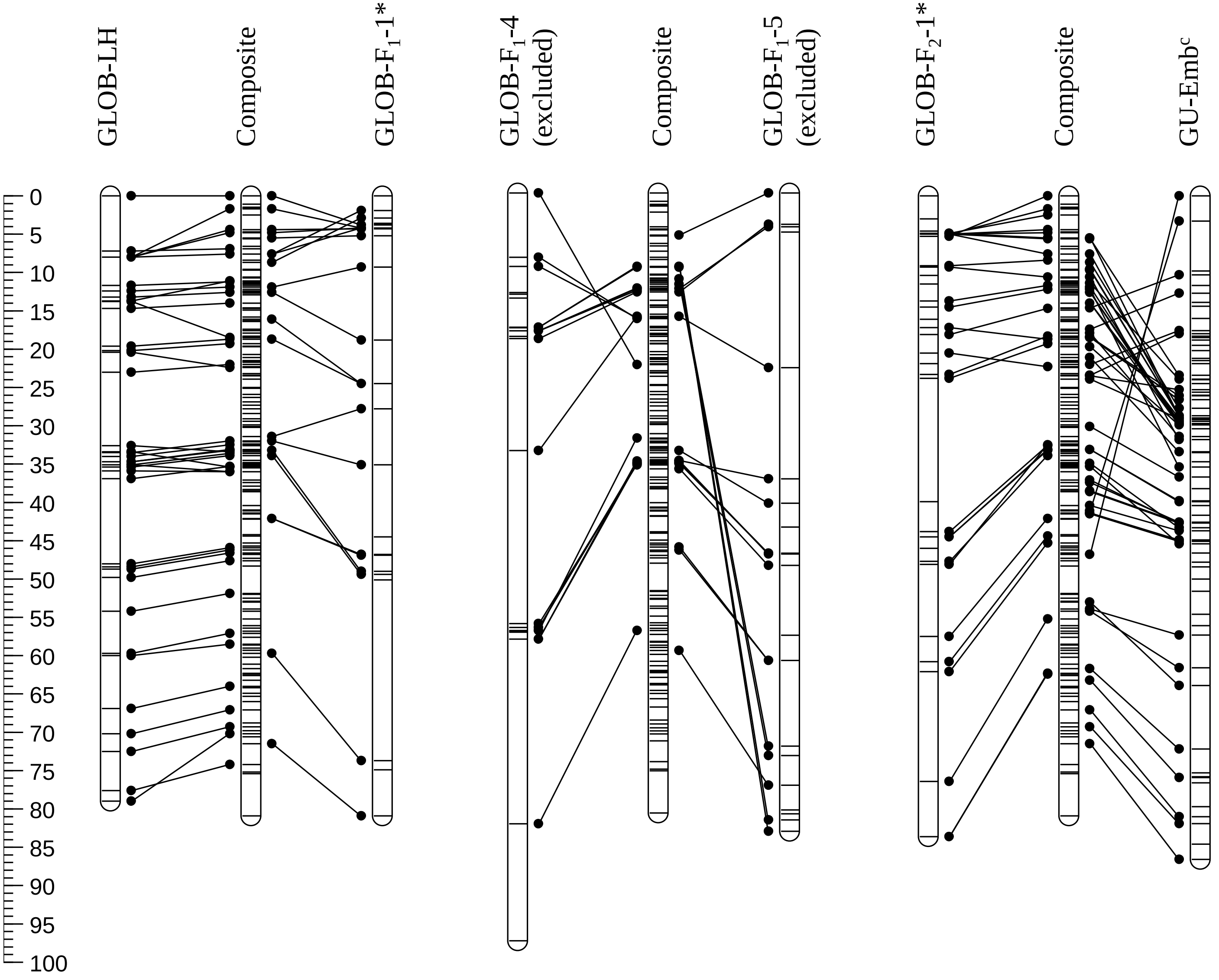

Linkage group 4

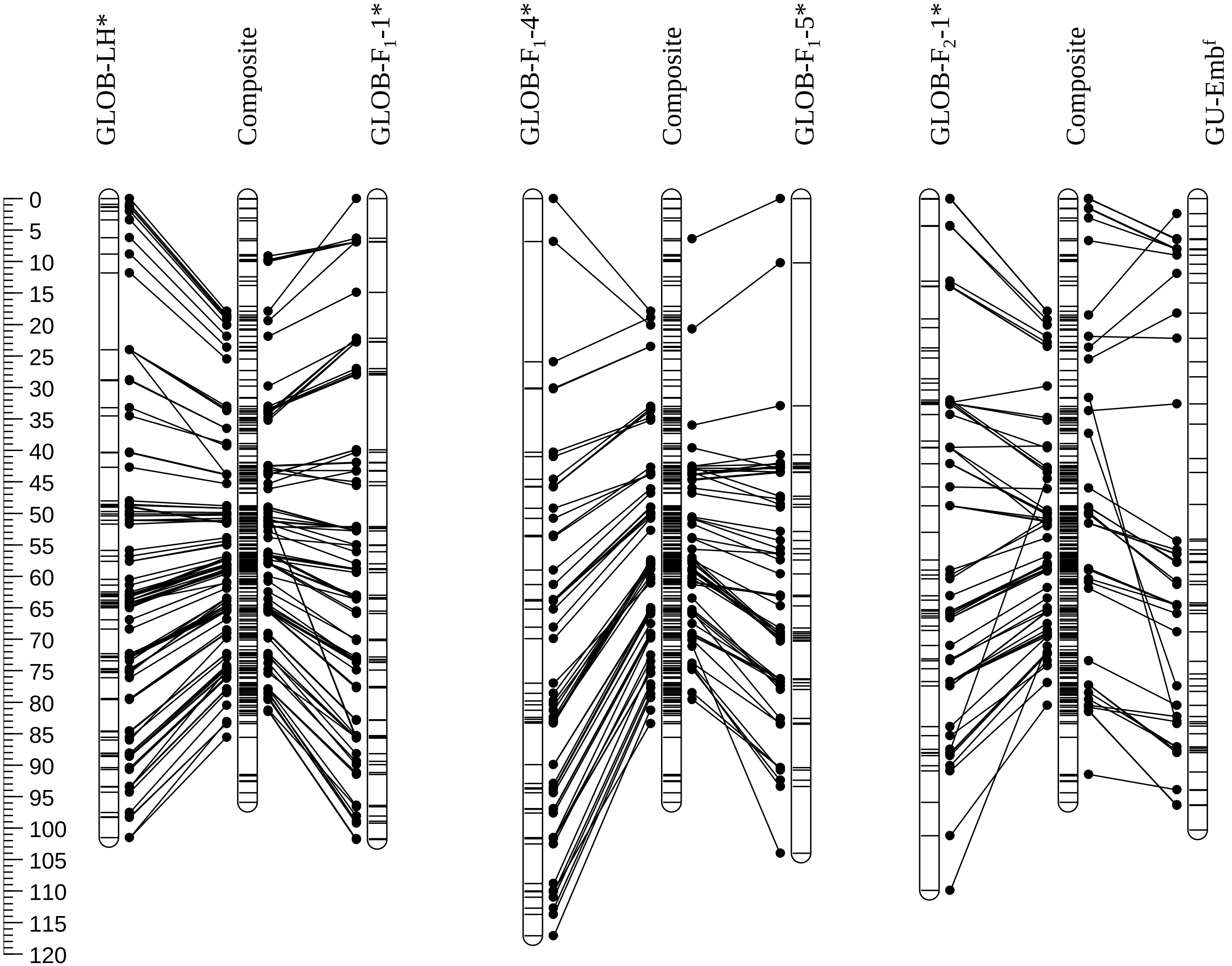

Linkage group 5

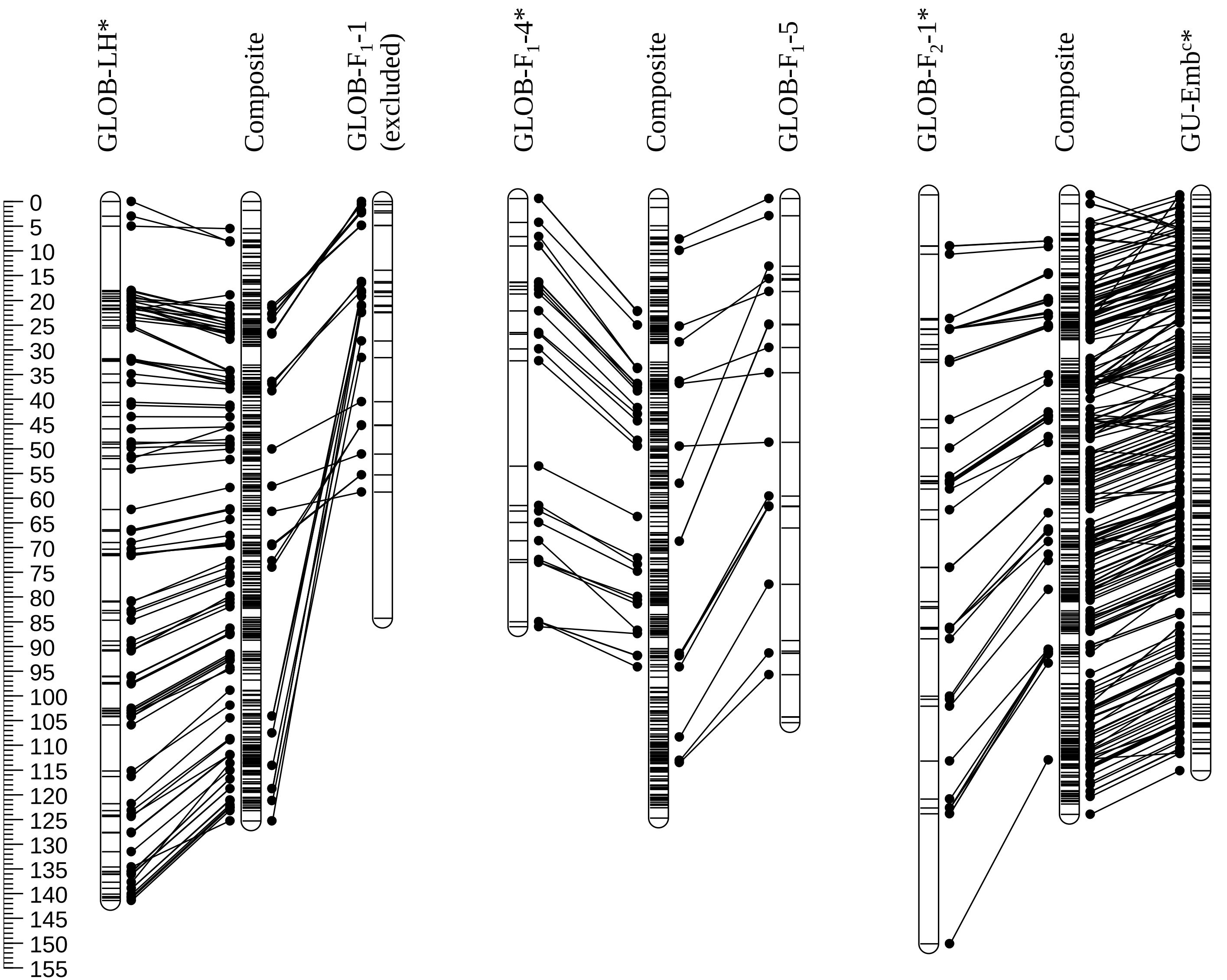

Linkage group 6

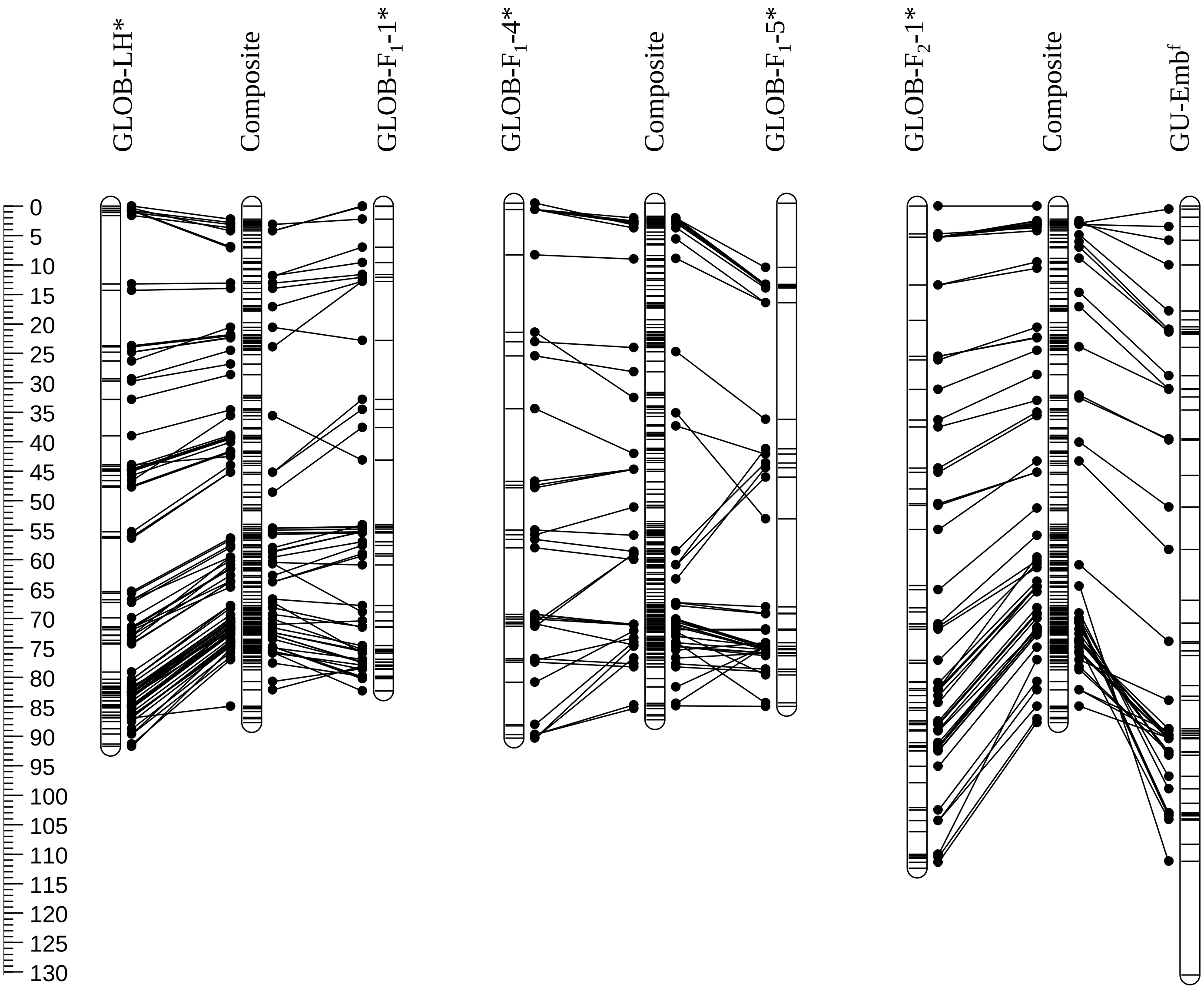

Linkage group 7

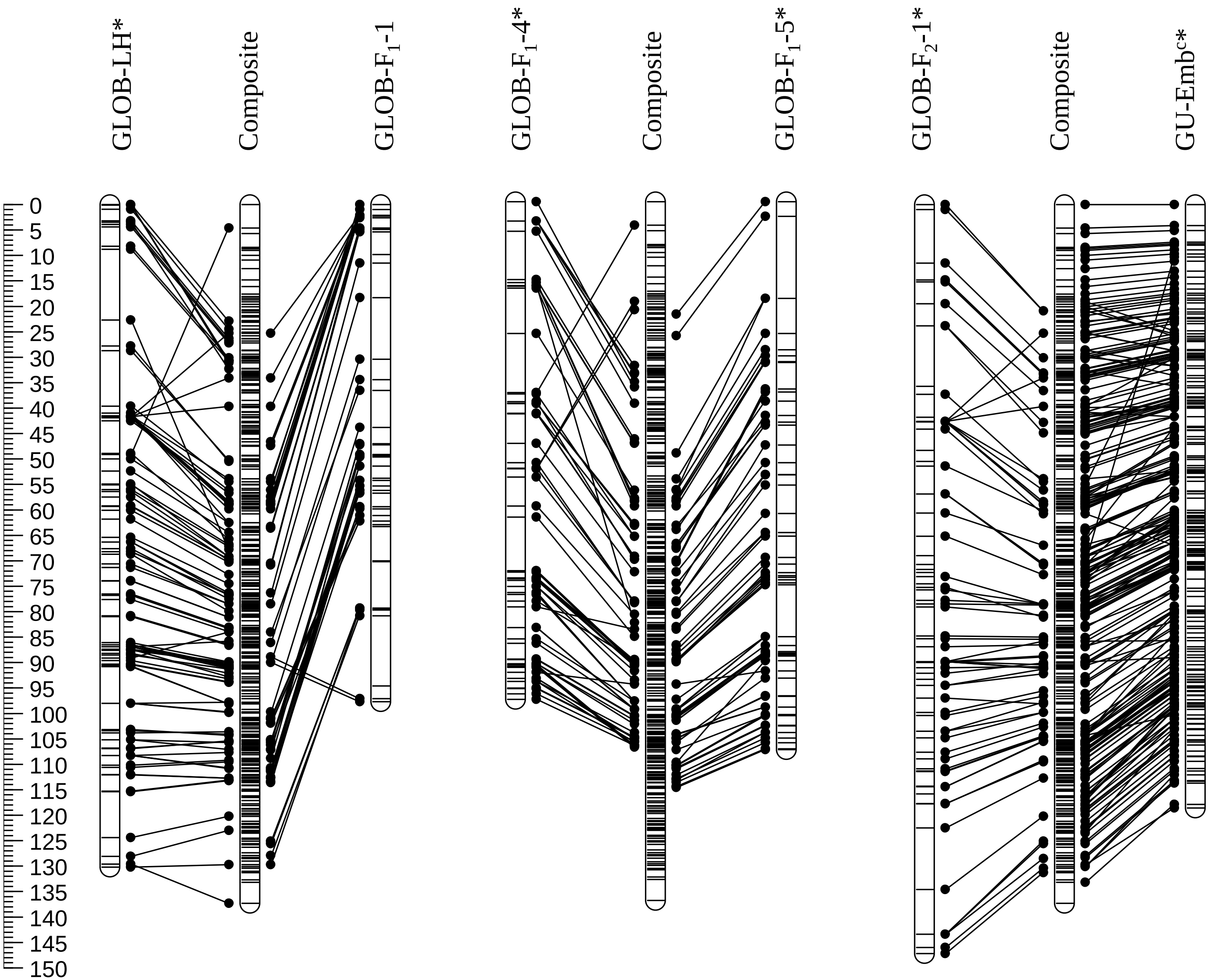

Linkage group 8

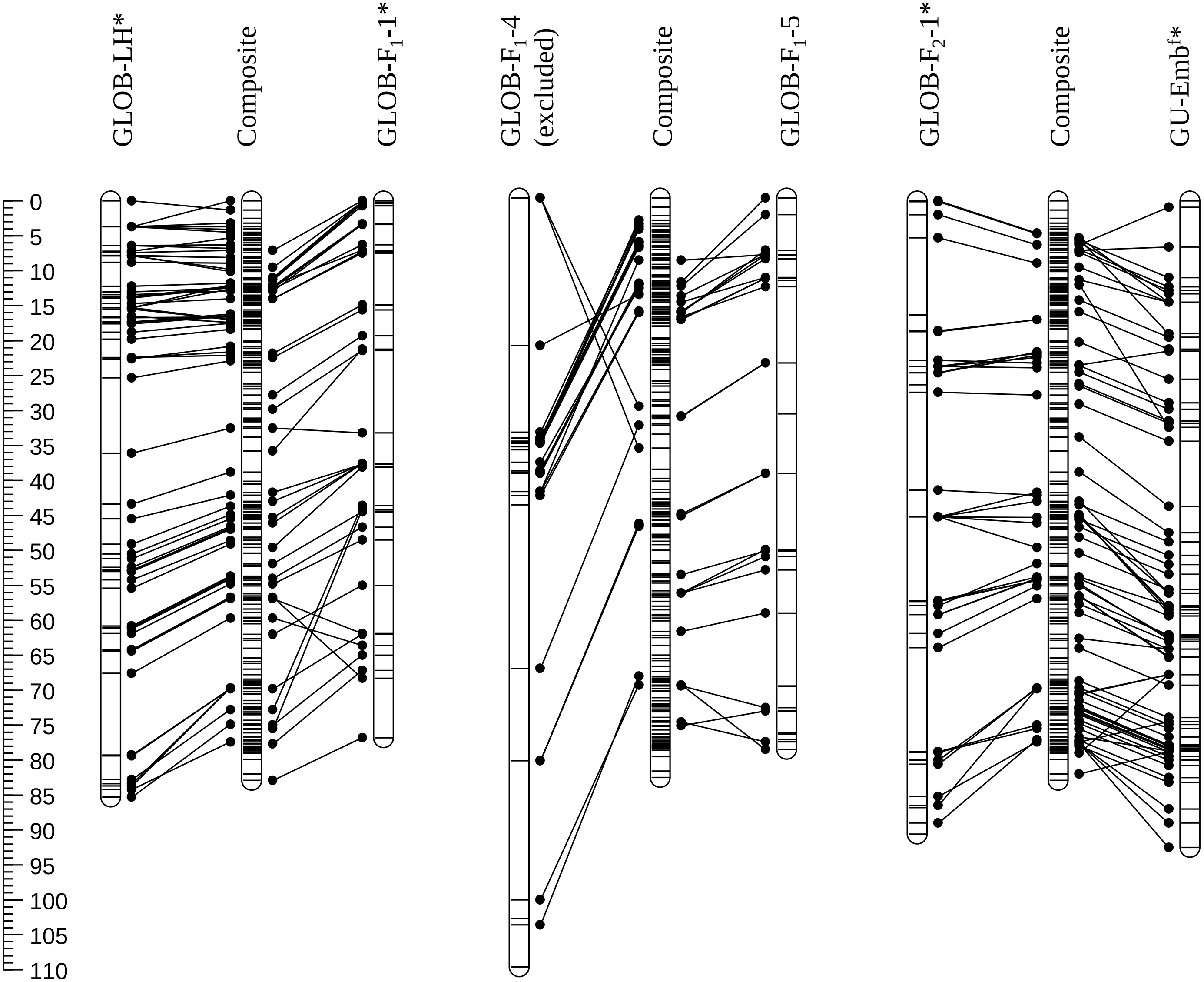

Linkage group 9

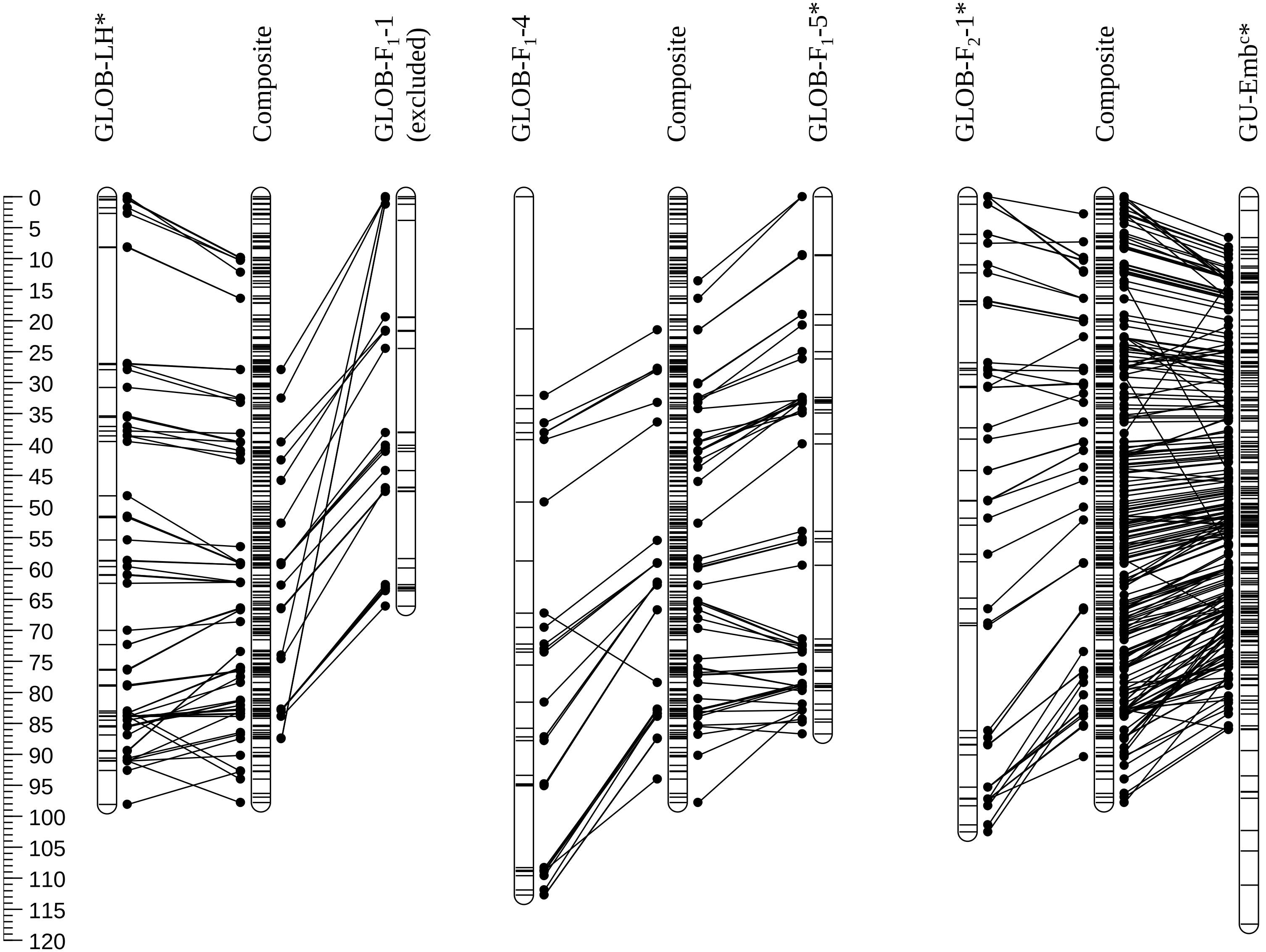

Linkage group 10

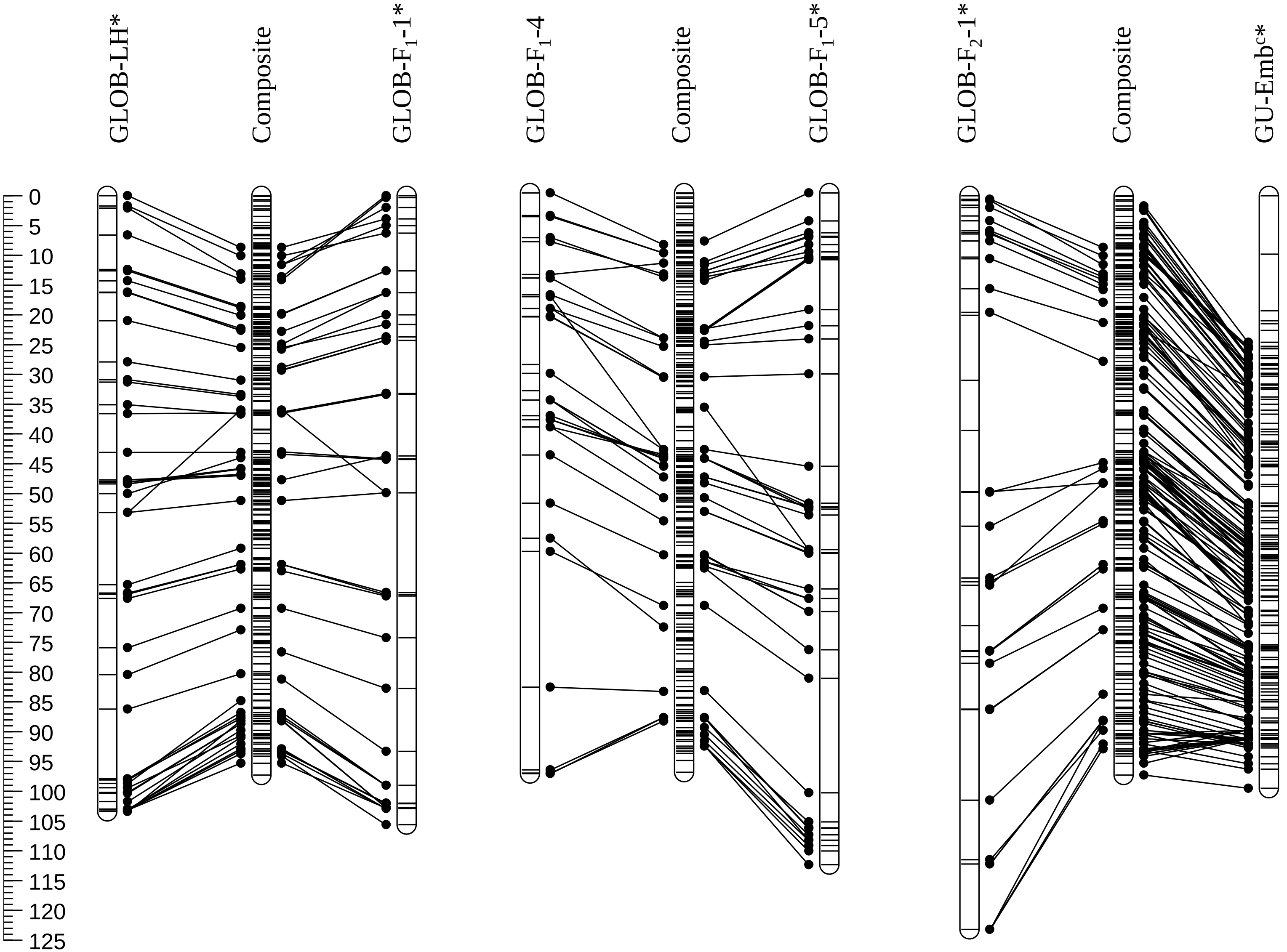

Linkage group 11
